# Improving the Quality of Ylang-Ylang Essential Oils [*Cananga odorata* (Lam.) Hook.f. &Thomson] Through Microwave-Assisted Extraction Compared to Conventional Extraction Methods

**DOI:** 10.3390/plants14203217

**Published:** 2025-10-20

**Authors:** Abacar Chakira, Christian Soria, Laura Lallemand, Gary Mares, Marc Chillet, Cyrielle Garcia

**Affiliations:** 1 CIRAD, UMR Qualisud, F-97410 Saint-Pierre, Réunion, France; chakira_abacar@yahoo.fr (A.C.); marc.chillet@cirad.fr (M.C.); 2 Qualisud, Univ Montpellier, Univ La Réunion, CIRAD, Institut Agro, Avignon Université, Montpellier, France; 3 LAR2SN, Faculté des Sciences et Techniques, Université des Comores, Moroni, Comoros; 4 CYROI, Plateforme de Recherche Cyclotron Réunion Océan Indien, F-97490 Sainte-Clotilde, Réunion, France; l.lallemand@cyroi.fr (L.L.); g.mares@cyroi.fr (G.M.)

**Keywords:** cananga odorata, solvent-free microwave extraction, hydrodistillation, steam-water distillation, volatile compounds

## Abstract

Solvent-free microwave extraction (SFME) is a clean and advanced method of extracting essential oils. In this study, it was compared to conventional hydrodistillation (HD) and steam-water distillation (SD), which are commonly used to extract essential oils from fresh ylang-ylang flowers. The yield and density of essential oils extracted via SFME within 40 min after the appearance of the first oil drop were higher than those obtained via conventional HD and SD within 3 h after the appearance of the first drop. Analysis of chemical compounds in the essential oils showed a high degree of variability in volatile compounds between the three extraction methods. Light oxygenated compounds are odor-active constituents. They comprised 81.23% of the SFME extract, whereas their levels were lower in the HD (69.94%) and SD extracts (57.98%). Total aromatic compounds were also higher in the essential oils obtained via SFME than in those obtained via HD and SD. These results support the use of SFME for ylang-ylang essential oil extraction, as it offers promising energy-/time-saving characteristics, along with higher quality.

## 1. Introduction

Essential oils (EOs) are usually extracted using physical methods, steam distillation (SD), hydrodistillation (HD), or solvent extraction [1,2]. On a commercial scale, steam distillation and hydrodistillation are the preferred methods of extracting EOs [2]. However, these methods have drawbacks in relation to decreased yield, with the loss of some volatile compounds or a low extraction efficiency; the degradation of initial compounds, particularly unsaturated or ester compounds as a result of thermal or hydrolytic effects; or the presence of toxic solvent residue in the extract [3,4,5]. These processes are also time- and energy-consuming [6].

These shortcomings have led to a search for alternative techniques of extraction, such as the use of supercritical fluids [7] and ultrasound [8] and microwave technology [9,10]. These advanced techniques are typically solvent-based and energy-efficient [11]. Supercritical fluid extraction is one of the best techniques for extracting EOs, but it is usually not used in small-scale industries due to its high cost [12,13]. As a result, microwave-assisted extraction techniques, which have lower set-up costs, have gained significant attention from researchers [14,15]. Microwave techniques can be used for EO extraction with or without the addition of water or an organic solvent. Adding a solvent can facilitate heating via microwaves; however, this approach carries a risk of hydrolyzing the EO due to excessive use of water as a medium [13]. In this context, solvent-free microwave extraction (SFME), one of the seven microwave extraction techniques reported in the literature, is an alternative option [16,17]. The vibration of water and other polar molecules during the microwave process causes an instantaneous increase in temperature and pressure inside plant cells and cell disruption through the evaporation of essential oils via azeotropic distillation, which may increase the yield of essential oils [18].

*Cananga odorata* (Lam.) Hook.f. & Thomson, commonly called ylang-ylang (forma *genuina*), is cultivated in order to obtain ylang-ylang EO from its fresh, ripe flowers. This EO has excellent olfactory richness and is particularly appreciated in luxury perfumery. It is also used in mass perfumery and the manufacture of cosmetics, detergents, deodorants, and soap. A wide range of chemical compounds are present in ylang-ylang EO, including bioactives with antimicrobial, antibiofilm, anti-inflammatory, antivector, insect-repellent, antidiabetic, and antimelanogenesis activities [19]. The commercial value and quality of ylang-ylang essential oils are mainly determined by their density [20,21]. The quality of an EO is also linked to its chemical composition, mostly in connection with the oxygenated compounds that govern aroma quality [13]. These compounds are highly odoriferous and hence the most valuable [22]. The chemical composition of the volatile secondary metabolites isolated from ylang-ylang depends, among other variables, on abiotic factors related to the geographical origin of the plant [23]; conditions before, during, and/or after essence extraction, such as the stage of flower development [24]; and the extraction method used [25,26].

In this study, microwave-assisted extraction is proposed as a novel technology for extracting ylang-ylang essential oil. A comparative study of SFME, hydrodistillation, and steam-water distillation was performed on ylang-ylang (forma *genuina*) for the first time. The quality of the resulting EOs were compared in terms of yield, density, and chemical composition.

## 2. Experiment

### 2.1. Plant Material

Fresh ylang-ylang flowers (*Cananga odorata*, forma *genuina*) were used in this study. Only undamaged, mature yellow flowers with a red-purplish heart were harvested. Sampling was conducted between January and February 2021 in the town of Saint-Pierre (Réunion Island; coordinates: −21.27724; 55.46689). This region is characterized by a tropical humid climate, with average daily temperatures of about 26 °C during the sampling period (25 °C min–27 °C max), and brown soil typical of the coastal plain. The flowers were obtained from traditionally managed trees cultivated without pesticide or fertilizer inputs. Freshly harvested flowers were immediately frozen in liquid nitrogen, stored at −80 °C in separate batches, and used for essential oil extraction. Extraction was performed in triplicate for each extraction method, using batches of 100 g of ylang-ylang flowers.

### 2.2. Essential Oil Extraction

The extractions were performed in a laboratory located at an altitude of 83 m above mean sea level (coordinates: −20.9065; 55.4987), with minimal variation in ebullition temperature compared to sea level (0.3 °C). The essential oils were brought into contact with anhydrous sodium sulfate salts before conditioning in amber glass vial under a nitrogen atmosphere. They were then maintained at 4 °C until analysis.

#### 2.2.1. Hydrodistillation Apparatus and Procedure

Hydrodistillation was performed using a Clevenger-type device, as previously described by Chakira et al. (2022) [24], on fresh flowers mixed with 1.5 L of distilled water.

#### 2.2.2. Steam-Water Distillation Apparatus and Procedure

A REUS hydrodistillation apparatus with a 12 L model vessel was used. Vegetable matter was distributed on dual perforated stainless steel grids above 1 L of water in the extraction vessel, which was subjected to electric heating. The stainless-steel cooling system continuously condensed the vapors, which were collected in a glass burette pre-filled with approximately 85 mL of distilled water, so that the amount of vapor was sufficient for the system to operate by recycling. Excess water was returned to the extraction vessel by cohobation. A LabTech recirculating water chiller (SMART H150-2100 model, Labtech, Hopkinton, MA, USA) was set at 8 °C.

#### 2.2.3. Solvent-Free Microwave Extraction Apparatus and Procedure

**Figure 1:**
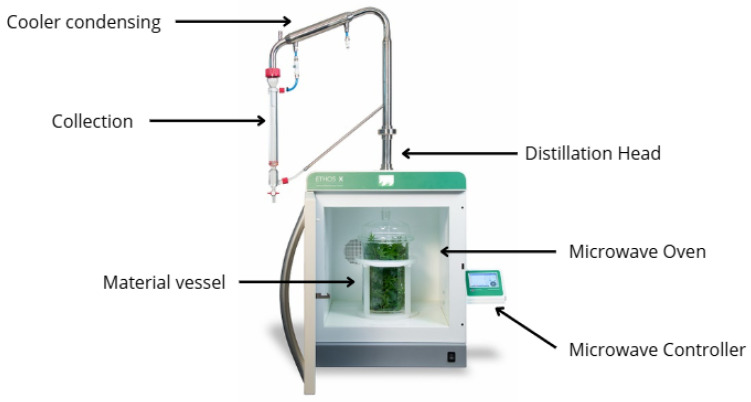
Schematic of microwave extraction apparatus.

A Milestone ETHOS X microwave steam extraction unit system (Milestone Srl, Sorisole, Italy) was used in fragrance/perfume mode (Figure 1). A batch of 100 g of flowers was placed in a 2 L glass microwave reactor and Teflon support (S model), without the addition of any solvent or water. The experiment was conducted at atmospheric pressure, and the power was set at 350 W and kept constant throughout the extraction process. The unit was connected to a cooling system identical to the one used in the SD setup. Internal heating of the water within the plant material causes the tissue to expand, leading to the rupture of glands and oleiferous receptacles. This process releases essential oils, which are then vaporized by the water inside the plant matter. The extraction time was set at 60 min for each batch. The temperature was continuously monitored using integrated sensors or through spot measurements and was maintained within 90 °C to 95 °C. Pressure was maintained at normal atmospheric pressure.

### 2.3. Density and Yield Measurements

Density was determined according to international standards [27].

The yield was then calculated by comparison with the vegetable matter (VM), using the following formula: yield = m (EO) × 100/m (VM), where m is expressed in grams.

### 2.4. GC-MS/FID Analysis

The samples were analyzed for their volatile compound composition using a Clarus580 SQ8 (PerkinElmer, Villebon sur Yvette, France), as previously described by Chakira et al. (2022) [24]. Briefly, 0.2 mL of EO was analyzed via mass spectrometry on an Elite-5MS Capillary Column in fused silica (PerkinElmer, USA) and via GC-FID on an Elite-FFAP column from PerkinElmer (Shelton, MT, USA). The oven temperature program was as follows: an initial temperature of 65 °C (held for 1 min) was ramped to 190 °C at 4 °C/min, increased to 285 °C at 15 °C/min, and maintained for 2 min. The injector and detector temperatures were set at 260 °C and 300 °C, respectively. Helium was used as the carrier gas at a constant flow rate of 0.6 mL/min. The injection volume was 0.2 mL, with the injector operating at 25,000 pts/s for a 1 μL injection. For mass spectrometry, the ionization energy was 70 eV, and mass spectra were acquired over the range *m*/*z* 30–400. The transfer line and ion source temperatures were 250 °C and 230 °C, respectively, with an electron multiplier voltage of 1568. A solvent delay of 6 min was applied, and the total run time was 74.5 min. Identification of volatile compounds with mass spectrometry was based upon the retention indices of the peaks, calculated from retention times using commercial C7-C30 n-alkane standards (49451 U, Supelco, Bellefonte, PA, USA). The retention indices were also compared with NIST MS database 17, using NIST MS search 2.3. Quantification results were expressed as the relative abundance with respect to all integrated peaks.

### 2.5. Statistical Analysis

Statistical analyses were performed using R.4.1.0/R Studio Statistical Software [28] with the packages openxlsx, ggplot2, ggpubr, multcomp, car, ggplot2, factoextra, FactoMineR, corrgram, corrplot, and missMDA. Statistical differences were assessed using Fisher’s test (*p*-value), Analysis of Variance (ANOVA), and Least Significant Differences (LSD). A Principal Component Analysis (PCA) was conducted to visualize correlations between variables and identify the homogeneity and heterogeneity of the different extraction methods.

## 3. Results and Discussions

### 3.1. Extraction Time, Yield, and Density

Table 1 lists the processing times, yields, and densities of the EOs extracted from ylang-ylang flowers by HD, SFME, and SD. The total duration of extraction after switching on the heating was set at 60, 226, and 230 min for the SFME, SD, and HD methods, respectively. The extraction temperature (indicated by the appearance of the first droplets of condensed oil), equivalent to the boiling temperature of water (100 °C), was reached in just 20 min using the SFME method, whereas 46 and 52 min were required when using the SD and HD methods, respectively, clearly demonstrating a time advantage of the SFME method. A similar observation was reported when using the stems, leaves, and flowers of *Origanum majorana*, for which the time to reach boiling temperature was three times shorter when using microwave-assisted extraction than when using HD and SD extraction [29]. A previous study reported that SFME enabled a considerable reduction in extraction time, down to 30 min compared to 60 min required for HD, with similar yields (around 0.48%) of EO. This shows that SFME is a shorter process that produces similar yields, costs less, and requires less solvent [30]. In the case of damask rose, to reach the extraction temperature (approximately 100 °C) and initiate evaporation of the first essential oil droplets with steam, the mixture required heating for 2.50 ± 0.29 min using SFME, compared to over 40 min using HD (42.66 ± 0.32 min) [31]. These results confirm that, in contrast to the conventional conductive heating procedures (HD and SD), the microwave method can quickly infuse products with heat and is able to increase the temperature of the entire sample at a faster pace [31,32].

The observed yield was significantly different according to the extraction method used. An extraction time of 40 min using SFME provided significantly higher yields (0.956%) than those obtained using HD (0.80%) and SD (0.463%) with an extraction time of 3 h each. The same tests show that the yield obtained via HD was significantly higher compared to the yield obtained with SD. The densities obtained using SFME (0.988) and HD (0.985) were significantly higher than the density obtained using SD.

The results obtained in this study confirm the influence of the extraction method in relation to the yield and density of ylang-ylang essential oils. To our knowledge, no study has reported the influence of extraction method on the density of EO obtained from flowers.

Our results are consistent with existing data from the literature on aromatic herbs. For example, Ragab et al. report that for *Origanum majorana*, microwave-assisted extraction with an extraction time of 60 min resulted in a better yield than HD and SD extraction within 3 h (0.8, 0.73, and 0.66%, respectively). It can be seen that yield (SFME) > yield (HD) > yield (SD), as in the present study [29]. Other authors observed that for lavender flowers, 6 min of extraction via microwave steam distillation provided yields comparable to those obtained after 30 min of SD extraction [16]. In Portugal, Araujo et al. extracted EOs from the flowers and aerial parts of *Thymus mastichina* and reported that the relative yield of EO obtained from flowers using SFME (3.1%) was higher than the yield obtained with (0.91%); however, for rehydrated aerial parts, the yield from HD (3.16%) was higher than the yields obtained by SFME (2.4%) and SD (2.04%), i.e., yield (HD) > yield (SFME) > yield (SD) [33]. The yield of damask rose EO obtained by SFME (0.056%) was significantly higher than the yield obtained by HD (0.033%) [31]. The higher yield and density observed with SFME, followed by HD and then SD, may be due to the effectiveness of the volatilization of oxygenated compounds. Abbas et al. reported that the yield obtained using HD (0.82%) significantly exceeded the yield following supercritical fluid extraction (0.45%). In this case, the higher yield may have been due to efficient volatilization of terpenoid components and thus enhanced oil recovery or the selectivity of the supercritical fluid extraction technique [34]. The higher yield of SFME compared to HD is due to internal atmospheric pressure, which promotes the swelling of oil glands and cell wall rupture, releasing EOs that evaporate spontaneously via azeotropic distillation [35,36,37,38].

According to these results, SFME is an energy-saving and highly efficient method in comparison to SD and HD. Additionally, the absence of solvent makes it an environmentally friendly method.

The official ISO standard [21] defines five commercial grades of ylang-ylang essential oil. Among the physicochemical specifications considered in this classification, density is the key official parameter, as actors in the value chain differentiate EOs on this basis. However, density alone is not sufficient to evaluate the overall quality and authenticity of an essential oil. Previous studies have highlighted the importance of chemical composition, particularly the proportion of oxygenated compounds, as a more reliable indicator of authenticity and sensory quality [26,39]. Other physicochemical properties, such as the refractive index, acid value, ester value, and optical rotation, are also determining factors. In this study, we focus on density and detailed GC-MS profiling. Indeed, density remains a useful complementary parameter, aligning with the ISO standard, while chemical composition remains the most discriminating factor for defining the quality and authenticity of ylang-ylang EO.

### 3.2. Chemical Families, Groups, Subgroups, and Oxygenated Compounds

Total aromatic compounds comprised the main chemical family present in the EO samples, at 72.34%, 65.01%, and 57.29%, for SFME, HD, and SD, respectively (Table 2). Although the statistical analysis (ANOVA and Tukey’s test) did not show significant differences, the SFME EO showed a tendency to be richer in total aromatic compounds compared to the EOs obtained by the two conventional methods, i.e., 15.05% more than for SD and 7.9% more than for HD. The aromatic compound content was also slightly higher in the EO obtained by the HD method, at 7.76% more than in the EO obtained via SD. GC-MS/FID analysis showed that terpenes constituted the second largest family in the chemical composition of the ylang-ylang EOs obtained by these three methods. The total terpene content comprised 38.1%, 29.37%, and 21.11% of the samples obtained by SD, HD, and SFME, respectively. Significant differences in total terpenes were observed between the SFME and SD methods, with 16.99% more total terpenes in the EO obtained via SD. Terpenes and aromatic compounds (phenylpropanoids) are the major constituents of EOs and are responsible for their biological properties [40]. No significant difference was observed for the total aliphatic derivatives (third family), which comprised 6.52%, 5.5%, and 4.57% of the EOs obtained by SFME, HD, and SD, respectively.

The results were grouped according to chemical compounds to assess the effect of the extraction method on the chemical composition of the ylang-ylang EO. The EOs obtained by different extraction methods show significant differences in the content of total esters and total terpene hydrocarbons. Total esters were the highest in the SFME-extracted oil, in which they represented 60.5% of the identified volatile compounds, while the content of terpene hydrocarbons was the lowest at 10.95%. The SD-extracted oil was found to contain the highest terpene hydrocarbon content at 27.12% and the lowest total ester content at 47.13%. The HD-extracted oil contained 53.72% total esters and 16.08% terpene hydrocarbons. Esters are one of the main chemical groups contributing to the characteristic aroma of ylang-ylang essential oil, with benzyl acetate identified as the most significant component [40,41,42,43]. In contrast, terpene hydrocarbons are reported to have minimal influence on the sensory properties of the oil [44,45]. Statistical analysis showed that the ylang-ylang flower EOs extracted using different methods were rather similar in their composition regarding the proportion of total alcohols (12.35%, 9.4%, and 10.01%), oxygenated terpenes (13.29%, 10.16%, and 10.98%), ether-oxides (13.16%, 14.02%, and 11.74%), phenols (3.68%, 4.06%, and 3.37%), nitrogen compounds (0.42%, 0.6%, and 0.4%), and aldehydes (0.44%, 0.37% and 0.15%).

**Figure 2:**
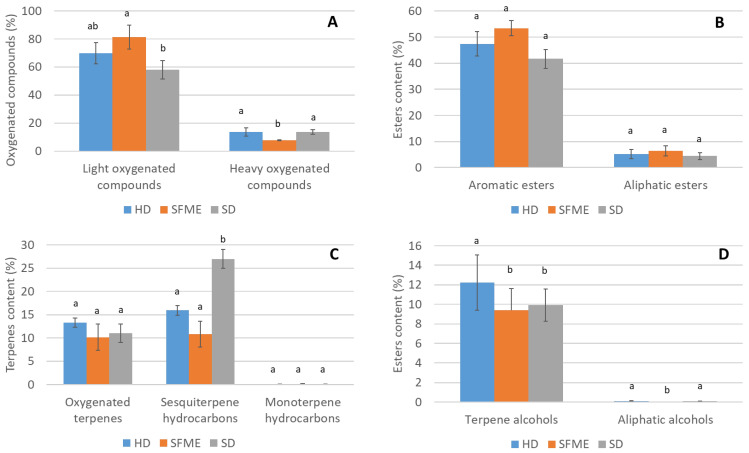
Proportion of main chemical groups in EOs obtained by HD, SFME, and SD extraction. (**A**) Oxygenated compounds, (**B**) esters, (**C**) terpenes and (**D**) alcohols. Different letters indicate significant differences (*p* ≤ 0.05).

Figure 2 shows the distribution of the principal chemical groups in the HD, SFME, and SD extracts.

More than 71%, 83%, and 88% of the compounds in the SD, HD, and SFME extracts were oxygenated compounds (Figure 2A). Among the EOs produced by the three extraction methods, the content of light oxygenated compounds, which are characterized by a low molecular weight (typically <150 g/mol), high volatility/low boiling point, and simple functional groups such as esters, alcohols, aldehydes, and ketones, was much higher than that of heavy oxygenated compounds, which are characterized by a high molecular weight (generally >150 g/mol), low volatility/high boiling point, and complex structures such as sesquiterpene alcohols, oxides, and lactones. The content of light oxygenated compounds in the oil obtained by SFME (81.23%) was significantly higher than in the oils obtained by SD (57.98%) and HD (69.94%), i.e., +23.24% and +11.29%, respectively. SFME provided more light oxygenated compounds, i.e., 35 compounds vs. 32 for HD and 30 for SD. Regarding heavy oxygenated compounds, HD and SD dominated in number and content, i.e., 14, 12, and 9 compounds were observed in the HD, SD, and SFME extracts, respectively. The EOs obtained via HD and SD both had a difference of +5.9% in heavy oxygenated compounds (13.69% and 13.68%) compared to the EO obtained by SFME (7.75%), but the SFME extract had the highest percentage of total oxygenated compounds overall, even if the difference was not significant.

The SFME method was particularly efficient for the extraction of oxygenated compounds and esters, as observed for light oxygenated compounds and aromatic esters (Figure 2B). The number of aromatic esters detected by GC-MS was consistent for each method, i.e., 13, 12, and 11 compounds detected in the EOs produced by SFME, HD, and SD respectively. No differences were observed in the levels of the aliphatic ester subgroup across extraction methods. The SD method was particularly effective at extracting terpenes, especially regarding sesquiterpene hydrocarbons in content (26.99% vs. 10.81% by SFME and 15.93%, by HD) but not in number of compounds (8 vs. 10 by SFME and 12 by HD). Similar low levels of monoterpene hydrocarbons were found in the HD, SFME, and SD extracts; likewise, no remarkable differences were observed in the oxygenated terpenes present in the EOs obtained using the different extraction methods (Figure 2C). The HD method appears to be more effective for the extraction of alcohol, notably terpene alcohols, with 12.23% compared to 9.4% and 9.92% from SFME and SD, respectively, and 12 identified compounds compared to 9 and 10 obtained from SFME and SD, respectively. The content of aliphatic alcohols was higher in the HD extract than the SD extract (0.12% and 0.09%, respectively). On the contrary, of the four aliphatic alcohols characterized by GC-MS/FID (3-buten-2-ol, 2-methyl-; 1-hexadecanol; prenol, and 2-octen-1-ol, 3,7-dimethyl-), none were detected in the SFME extract (Figure 2D).

The properties of ylang-ylang essential oil derive from its terpenes and oxygenated compounds. Oxygenated compounds are of greater importance than monoterpenes in terms of this essential oil’s distinctive smell [46]. Indeed, light and heavy oxygenated compounds are considered quality markers in ylang-ylang oil [26,47,48]. Therefore, we can assume that for ylang-ylang essential oil, SFME has an advantage in terms of perfume aroma when compared to HD and SD methods.

Bendahou et al. (2008) compared SFME and conventional HD of *Origanum glandulosum* Desf, reporting a considerable increase in the amount of total oxygenated compounds in the SFME extract (87.4%) compared to the HD extract (45.6%) and a significant decrease in total non-oxygenated compounds (hydrocarbons) in the SFME extract (10.1%) compared to the HD extract (54.2%) [49]. These results are similar to those observed in this study (Table 3) and another showing a higher proportion of oxygenated compounds in *Ocimum basilicum* L., garden mint *(Mentha crispa* L.), and thyme *(Thymus vulgaris* L.) EOs extracted by SFME (82.2%, 69.6%, and 77.6%) compared to those extracted by HD (63.7%, 67.1%, and 53.1%) [1]. Higher amounts of oxygenated compounds and lower amounts of monoterpene hydrocarbons are frequently reported in EOs extracted by SFME compared to those extracted by HD. This might be explained by the presence of oxygen, which is the second most electronegative atom and often undergoes polarization of its molecular bonds. In effect, compounds with high and low dipolar moments (compounds with at least one polarized bond for which the barycenter of the negative charges does not coincide with the barycenter of the positive charges) can be differentially extracted, depending on their dipole moment. Organic compounds with higher dipole moments exhibit stronger interactions with the microwave field due to the intensity of their positive and negative charges and can be extracted more easily [18]. The proportion of oxygenated compounds in SFME extracts is higher due to the higher dipolar moments in these organic compounds, which facilitate their extraction, in contrast to monoterpene hydrocarbons, which have lower dipolar moments [18]. Shiwakoti et al. (2017) reported a similar observation regarding terpene hydrocarbon [50]. In their study, the SD method demonstrated superior performance compared to HD in the extraction of hydrocarbons from basil essential oil. This outcome is explained by the higher heat transfer in SD vapors than in HD solubilization. Direct contact of the plant with water vapor carries volatile material, resulting in rapid elution of the compounds and, consequently, higher accumulation [50].

### 3.3. Diversity of Volatile Compounds in Essential Oils Obtained by HD, SFME, and SD

**Figure 3:**
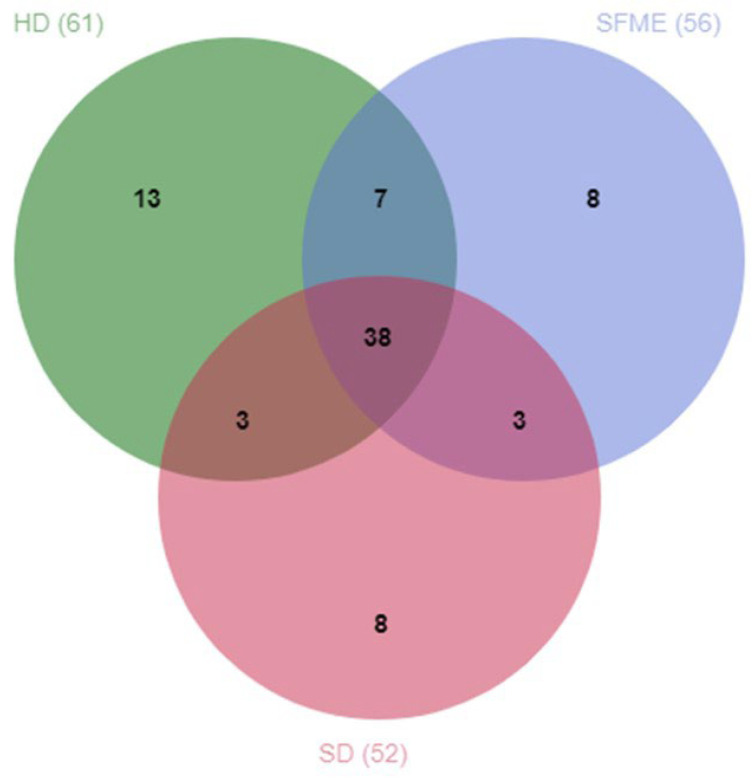
Jvenn diagram showing the number of specific or shared compounds, grouped by HD, SFME, and SD methods of extraction.

The Jvenn diagram (Figure 3) below shows the number of volatile compounds detected in EOs obtained by each method, as well as the compounds shared between two or three methods. More volatile compounds were detected in the HD extract compared to the SFME and SD extracts (Figure 1). In the EOs obtained by HD, SFME, and SD, 61, 56, and 52 compounds were detected, respectively, of which 38 are common to all three extracts. Among the 13 compounds specific to the HD extract and the 8 specific to the SD extract, a majority were alcohols, even though this category had more representation in the HD EO than the SD EO. The eight SFME-specific compounds were mainly esters and phenols.

### 3.4. Chemical Composition

The volatile compounds identified in the EOs extracted by HD, SFME, and SD comprised 99.92%, 99.97%, and 99.96% of the total samples, respectively. For aromatic compounds, 20, 23, and 24 volatile compounds were detected in the EOs obtained by SD, HD, and SFME, including 11, 12, and 13 esters; 3, 3, and 5 phenols; 2, 3, and 2 ether-oxides; 3, 3, and 2 nitrogen compounds; 1, 1, and 2 aldehydes and 0, 1, and 0 alkenes, respectively. Benzyl acetate, benzene methanol-2-methyl acetate, benzene methanol-4-methoxy-, acetate, *p*-cresol, acetyl eugenol, and 2-phenyl nitroethane were the aromatic compounds most influenced by the SFME method, i.e., they were significantly higher in the EO obtained by SFME than in the other extracts (SD and HD). In alignment with this, only phenylethyl acetate was strongly affected by HD, and none were influenced by SD (Table 2). GC-MS/FID analysis facilitated the characterization of 28, 23, and 23 terpene compounds in the EOs extracted by HD, SFME, and SD, including 14, 12, and 10 hydrocarbons; 12, 9, and 10 alcohols; 1, 1, and 2 esters; and 1 oxide for each extraction method, respectively. The compounds influenced in the largest proportion by the extraction methods were as follows: α-copaene, α-farnesene, germacrene-D, germacrene-D-4-ol, alloaromadendrene, guaiol, epi-cubenol, and agarospirol for SD; α-muurolene, junenol, caryophyllene alcohol, 10-epi-γ-eudesmol, and bulnesol for HD, and no compounds for SFME. The aliphatic derivatives detected in the EOs obtained by HD (10), SFME (9), and SD (9) consisted of esters (6, 7, and 6), alcohols (2, 0, and 2), and aldehydes (2, 2, and 1). The extraction of prenyl acetate and that of (E, E)-farnesyl acetate were strongly influenced by SFME, which produced an EO with the highest content of the former and the lowest of the latter. 1-butanol, 3-methyl-, acetate, and 3-methyl-3-butene-1-ol acetate were influenced much more by the SD extraction method.

As shown in Table 2, the main compounds (≥5%) in the EOs obtained by HD, SFME, and SD were benzyl acetate, *p*-cresyl methyl ether, methyl benzoate, linalool, (E, E)-cinnamyl acetate, benzyl benzoate, α-farnesene, and germacrene-D. These compounds were also the main components found in previous studies of ylang-ylang EO (forma *genuina*) [21,26,39,42,47,48]. Furthermore, these 8 compounds all belong to the 15 volatile compounds considered characteristic of ylang-ylang forma *genuina* EO by the French standard NF-ISO 3263 [21]. Notably, we found that 6/8 were significantly influenced by the mode of extraction. A comparison of the characteristics of the EOs obtained in this study and the international standards for ylang-ylang EO was achieved (Table A1). According to density, the EOs obtained by HD (0.985) and SFME (0.988) belong to the “Extra superior” category, while the EO obtained by SD (0.959) belongs to the Extra/First/Second fractions. The content of the 15 marker compounds is overall consistent with the AFNOR specifications, despite some overlap between categories and with the exception of β-caryophyllene, which was not quantified in this work. The NF ISO 3063 standard [21] was established in 2005 for oils from Comoros and Madagascar, and slight compositional shifts may occur over time due to climate change. Overall, these results support alignment with the AFNOR categorization and confirm the quality and comparability of the oils studied.

The results of the EO analysis indicated considerable variations in EO compounds extracted using the SFME method compared with the HD and SD methods. Also, 35 more or less abundant compounds (0.1–5%) were influenced by the three extraction methods used (HD, SFME, and SD). Statistical analysis shows that in the EOs produced using all methods, 64 volatile compounds out of the 80 characterized by GC-MS/FID were influenced by the extraction method, i.e., 80% of the sample. This chemical variation may be the result of the thermal and hydrolytic degradation of the compounds by oxidation or trans-esterification due to the temperature and extraction time applied in the method [38,51]. Hydrolytic and thermal processes may alter oxygenated monoterpenes, potentially leading to shifts in the balance toward monoterpene hydrocarbons [37]. This occurred with α-pinene, β-pinene, and camphene in the degradation of the chemical composition of basil essential oils isolated by HD and SFE, which increased with the HD method, possibly due to the detrimental effect of oxygenated compounds [34]. In this study, α-pinene and cubebene content increased with the HD method, but β-pinene and camphene were not detected.

Compared to the traditional distillation methods, the oils obtained by SFME were rich in aromatic compounds (esters) and light oxygenated compounds, such as benzyl acetate and E-cinnamyl acetate, which may positively influence bioactivity due to the antioxidant and antimicrobial properties of these molecules [41,42,43,52]. Our findings are consistent with previous reports indicating that SFME enhances both the yield and quality of essential oils compared to conventional techniques. Chenni et al. [30] also showed that basil oils obtained by SFME contained higher levels of oxygenated constituents with strong antimicrobial activity compared to oils obtained by hydrodistillation. Similarly, Bendahou et al. [49] reported the selectivity of SFME for thymol in Origanum glandulosum, while Villa et al. [53] found that extracting Rosa damascena via SFME produced essential oils richer in benzyl alcohol and 2-phenylethanol, both of which are desirable fragrance components. Bioactivity testing would help clarify the impact of extraction method on these properties.

In our study, oxygenated odor-active compounds accounted for over 80% of the SFME extracts, compared to much lower proportions following hydrodistillation and steam distillation, confirming the ability of SFME to better preserve key aroma constituents. From a sensory perspective, the higher proportion of aromatic esters in the oils obtained by SFME should directly affect the characteristic aroma of ylang-ylang essential oil. In particular, benzyl acetate and *p*-cresyl methyl ether may contribute to a more pronounced and complex fragrance [41,42,43,52]. These effects need to be confirmed through olfactory measurements. Terpene hydrocarbons were present in lower quantities in the SFME extract, and these molecules, particularly sesquiterpenes and monoterpenes, play a role in the complexity and depth of the scent.

Some limitations of this study should be acknowledged. The efficiency of SFME depends on the moisture content and dielectric properties of the raw material, which can affect reproducibility across batches. Certain highly volatile or thermolabile compounds may undergo partial degradation or be lost during microwave irradiation, as reported in other aromatic plants [53,54]. Further process optimization may be beneficial, especially if the goal is to tailor the sensory profile for specific applications. For example, variations in microwave power and extraction time could achieve a balance between maximizing the content of desirable aromatic esters while preserving terpene levels, which would improve the overall sensory complexity of the oil.

### 3.5. Principal Component Analysis of Ylang-Ylang Essential Oils Obtained by HD, SFME, and SD

**Figure 4:**
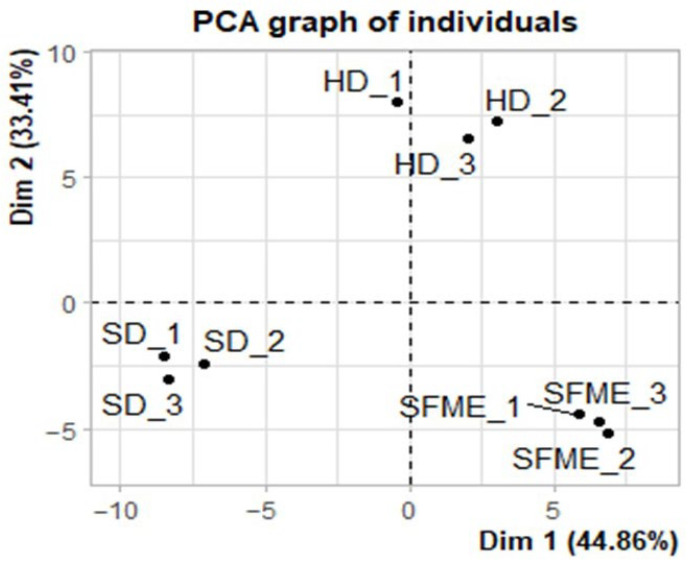
Principal component score chart of the three methods of extracting essential oil from ylang-ylang flowers.

A principal component analysis by covariance matrix was performed on the total volatile compounds in the essential oils obtained by HD, SFME, and SD, which were characterized by GC-MS/FID (Figure 4). The analysis was intended to confirm whether the chemical composition of the essential oils varied between the different methods used. The variance contribution rates of PC1 (principal component 1) and PC2 were 44.86% and 33.41%, respectively, presenting a total variance of the original data of 78.27%. The distribution of individuals between the two dimensions showed that the samples of three extraction methods are relatively independent. This confirms the existence of high chemical variability between essential oils extracted by HD, SFME, and SD.

**Figure 5:**
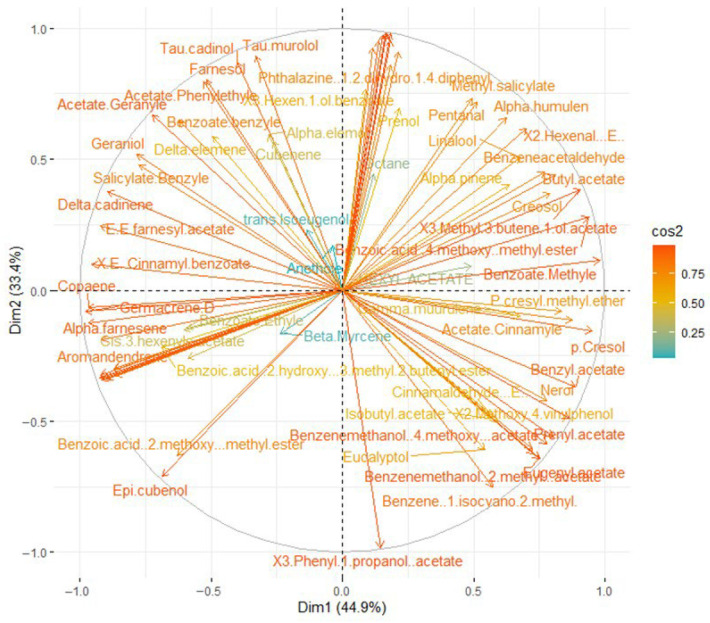
Principal component analysis of total volatile compounds identified in essential oils extracted using the three methods.

Figure 5 below shows that (E.E)-α-farnesene, germacreneD, α-copaene, aromandendrene, 3-buten-2-ol, 2-methyl acetate, (E)-cinnamyl benzoate, (E,E)-farnesyl acetate, and δ-cadinene were associated with the first principal component (PC1) in the negative score and showed a positive correlation with the SD method. Among them, the high presence of hydrocarbon sesquiterpenes like (E.E)-α-farnesene and δ-cadinene is notable—these compounds create the base of the warm notes onto which other molecules can be grafted. Benzyl acetate, E-cinnamyl acetate, acetyl eugenol, *p*-cresyl methyl ether, *p*-cresol, and nerol prenyl acetate were projected to axis 1 in the positive score and were highly positively correlated with the SFME extraction method. The notable presence of ethers like *p*-cresyl methyl ether, phenols like *p*-cresol, and esters like benzyl acetate indicate a powerful smell. Indeed, the first group are responsible for the medicinal, diffuse, and penetrating scent of ylang-ylang EO; the second group confer characteristic spicy and balsamic warm notes; and the third compound is a major contributor to the typical scent of ylang-ylang [24]. Therefore, use of SFME, yielding high concentrations of these compounds, offers a powerful smell and higher quality extract for perfumery. In contrast, volatile compounds such as α-humulene, linalool, methyl salicylate, and phenylethyl acetate were strongly associated with the second principal component (PC2) and positively correlated with the HD method. Linalool is linked to fruity, spicy notes, while methyl salicylate is also related to ylang-ylang’s typical smell, probably resulting in the fruity smell of these extracts. The distribution of individuals between the two dimensions showed that the samples obtained by the three extraction methods are relatively independent, illustrating the high chemical variability between essential oils obtained by HD, SFME, and SD.

In addition to yield and compositional improvements, the industrial application of SFME deserves attention. SFME is widely recognized as a green extraction technology, offering reduced energy use, extraction time, and solvent consumption while maintaining or improving oil quality compared to conventional HD and SD [53,54]. This technique minimizes thermal degradation, leading to better preservation of oxygenated compounds and delicate volatiles that are often degraded or lost under conventional processes [55,56]. This makes SFME particularly valuable for high-end industrial applications in perfumery, cosmetics, and aromatherapy, where aroma fidelity and compound stability are critical [26,53].

From a scalability perspective, SFME has been successfully adapted to semi-industrial setups, confirming its feasibility for processing larger volumes of biomass with reproducible yields and chemical profiles [54,56]. In ylang-ylang and other aromatic plants, this method not only accelerates production but also generates oils with enhanced antimicrobial, antioxidant, and preservative properties, reinforcing their industrial potential for food preservation and cosmetic formulations [53,57]. Furthermore, SFME aligns with sustainability goals, as its reduced energy demand and lower CO_2_ emissions support cleaner production pathways [54].

However, some drawbacks persist. The high capital cost of microwave reactors compared to conventional systems remains a major barrier to adoption in small-scale production systems, particularly in developing countries [53]. Efficient extraction requires careful parameter optimization to avoid batch-to-batch variability [55,56]. Another limitation is that although pilot-scale demonstrations are encouraging, continuous large-scale processing still requires more techno-economic validation [54].

Overall, SFME is a promising industrial alternative for the extraction of high-value essential oils such as ylang-ylang, combining rapidity, energy savings, and improved oil quality. However, its broader industrial adoption will depend on reducing the cost of the equipment, process standardization across raw materials, and further validation under full-scale industrial conditions [26,53,56].

## 4. Conclusions

Ylang-ylang essential oils were extracted using advanced SFME and compared with essential oils extracted using conventional HD and SD techniques. The essential oils’ chemical compounds, yield, density, and extraction times were analyzed. The results indicate solvent-free microwave extraction is the best extraction technique. Indeed, with SFME, the maximum yield and density were reached in only 40 min, which can be compared to 3 h for HD and SD. Additionally, SFME resulted in a higher content of oxygenated and aromatic compounds, esters, phenols, and ether-oxides. HD was superior to SD in terms of yield, density, and oxygenated compound, alcohol, and ester content. The higher rate of extraction of light and heavy oxygenated compounds in SFME suggest an advantage for ylang-ylang aroma extraction for the perfumery industry. Further analysis may confirm the impact of SFME on key odorant molecules.

Upscaling studies are needed to ensure the feasibility of this technique at an industrial scale; some challenges remain [58] in relation to the possibly inconsistent distribution of temperature over a larger sample volume, radiation leakage, and the design of upscaled units. Financial assessment is required to determine process viability.

## Figures and Tables

**Table 1:** Processing time, yield, and density of essential oils obtained from ylang-ylang flowers by HD, SFME, and SD.

Extraction Method	Processing Time (min)	Yield (%)	Density
SFME	60 (20 + 40) ± 0	0.956 ± 0.06 ^a^	0.988 ± 0.002 ^a^
HD	232 (52 + 180) ± 2	0.80 ± 0.01 ^b^	0.985 ± 0.004 ^a^
SD	226 (46 + 180) ± 1	0.463 ± 0.02 ^c^	0.959 ± 0.003 ^c^

Values are means ± standard errors of means and were averages of three extractions per method. Means followed by a different letter within the lines are significantly different (*p* ≤ 0.05), Tukey’s test.

**Table 2:** Chemical composition of ylang-ylang essential oils extracted by HD, SFME, and SD.

Chemical Family	Chemical Group	RT [min]	RI	Component Name	Extraction Method		
					HD	SFME	SD
Total aromatic compounds					65.01 ± 4.61 ^a^	72.34 ± 6.07 ^a^	57.29 ± 3.85 ^a^
	ester	19.07	1262	benzyl acetate	20.14 ± 1.79 ^a^	28.94 ± 2.44 ^b^	15.98 ± 1.59 ^a^
	ester	28.02	1852	benzyl benzoate	7.43 ± 1.36 ^a^	4.6 ± 0.29 ^b^	7.08 ± 0.98 ^a^
	ester	29.47	1952	benzyl salicylate	3.16 ± 0.77 ^a^	1.73 ± 0.22 ^b^	4.6 ± 0.29 ^a^
	ester	18.21	1210	methyl benzoate	8.39 ± 0.63 ^a^	9.01 ± 0.06 ^a^	6.23 ± 0.17 ^b^
	ester	23.18	1521	(E,E)-cinnamyl acetate	7.48 ± 0.39 ^a^	7.84 ± 0.05 ^a^	7.06 ± 0.14 ^a^
	ester	19.27	1275	ethyl benzoate	0.07 ± 0.02 ^a^	0.06 ± 0.01 ^a^	0.1 ± 0.04 ^a^
	ester	19.72	1302	methyl salicylate	0.34 ± 0.04 ^ab^	0.29 ± 0.01 ^bc^	0.26 ± 0.01 ^c^
	ester	23.20	1522	phenol, 4-(2-propenyl), acetate	-	0.05 ± 0.01 ^a^	-
	ester	20.39	1344	phenyl ethyl acetate	0.32 ± 0.05 ^a^	-	0.24 ± 0.02 ^c^
	ester	22.24	1460	benzoic acid, 2-methoxy, methyl ester	-	0.04 ± 0.01 ^b^	0.07 ± 0.02 ^c^
	ester	20.70	1363	benzoic acid, 4-methoxy, methyl ester	0.09 ± 0.01 ^a^	0.09 ± 0.01 ^a^	-
	ester	20.96	1380	benzene methanol, 2-methyl, acetate	-	0.57 ± 0.03 ^a^	-
	ester	23.26	1526	benzene methanol, 4-methoxy, acetate	0.02 ± 0.03 ^ac^	0.15 ± 0.01 ^b^	-
	ester	22.07	1449	3-phenyl-1-propanol, acetate	-	0.03 ± 0 ^b^	0.02 ± 0 ^c^
	ester	31.97	2110	(E)-cinnamyl benzoate	0.01 ± 0 ^a^	-	0.02 ± 0.01 ^c^
	ester	24.97	1640	3-hexen-1-ol, benzoate, (Z)	0.03 ± 0.02 ^a^	-	-
	phenol	23.29	1528	isoeugenol	3.25 ± 0.3 ^a^	3.14 ± 0.25 ^a^	3.19 ± 0.06 ^a^
	phenol	17.69	1178	*p*-cresol	0.4 ± 0.08 ^a^	0.61 ± 0.04 ^b^	0.17 ± 0.02 ^c^
	phenol	19.54	1291	creosol	0.03 ± 0.01 ^a^	0.03 ± 0.01 ^a^	0.01 ± 0 ^b^
	phenol	25.74	1692	acetyl eugenol	-	0.27 ± 0.02 ^a^	-
	phenol	21.98	1444	2-methoxy-4-vinylphenol	-	0.01 ± 0.01 ^a^	-
	ether-oxide	17.13	1143	*p*-cresyl methyl ether	12.62 ± 1.46 ^a^	13.54 ± 0.24 ^a^	11.23 ± 0.93 ^a^
	ether-oxide	20.96	1380	anethole	0.52 ± 0.11 ^a^	0.48 ± 0.05 ^a^	0.51 ± 0.07 ^a^
	ether-oxide	18.34	1218	dimethoxy-1,2-benzene	0.02 ± 0.01 ^a^	-	-
	nitrogen compound	21.24	1397	2-phenyl nitroethane	0.37 ± 0.03 ^a^	0.58 ± 0.04 ^b^	0.37 ± 0.02 ^a^
	nitrogen compound	18.82	1247	benzene, 1-isocyano-2-methyl	0.01 ± 0.0 ^a^	0.02 ± 0 ^b^	0.01 ± 0 ^a^
	nitrogen compound	32.61	2145	1-benzylindole	-	-	0.02 ± 0.01 ^b^
	aldehyde	17.49	1165	benzeneacetaldehyde	0.28 ± 0.07 ^a^	0.24 ± 0.04 ^a^	0.12 ± 0.02 ^b^
	aldehyde	20.31	1339	cinnamaldehyde, (E)	-	0.02 ± 0.01 ^b^	-
	alkene	21.89	1438	benzene, 1-propenyl	0.03 ± 0 ^a^	-	-
Total terpenes					29.37± 1.90 ^abc^	21.11± 1.57 ^b^	38.1± 2.90 ^c^
	monoterpene hydrocarbon	16.04	1076	*α*-pinene	0.12 ± 0 ^a^	0.10 ± 0.03 ^a^	0.09 ± 0.01 ^a^
	monoterpene hydrocarbon	16.43	1100	β-myrcene	0.03 ± 0.01 ^a^	0.03 ± 0 ^a^	0.04 ± 0.02 ^a^
	sesquiterpene hydrocarbon	22.51	1477	*α*-copaene	0.19 ± 0.03 ^a^	0.14 ± 0.02 ^a^	0.34 ± 0.04 ^b^
	sesquiterpene hydrocarbon	21.72	1427	*δ*-elemene	0.15 ± 0.01 ^a^	0.12 ± 0.01 ^a^	0.15 ± 0.02 ^a^
	sesquiterpene hydrocarbon	23.32	1530	*α* -humulene	0.29 ± 0.02 ^a^	0.16 ± 0.11 ^a^	-
	sesquiterpene hydrocarbon	23.88	1567	*α*-farnesene	7.15 ± 0.45 ^a^	5.68 ± 0.94 ^a^	12.61 ± 1.2 ^b^
	sesquiterpene hydrocarbon	24.07	1579	germacrene-D	5.8 ± 0.38 ^a^	3.55 ± 1.14 ^b^	12.24 ± 0.93 ^c^
	sesquiterpene hydrocarbon	24.46	1605	*δ*-cadinene	0.87 ± 0.06 ^a^	0.45 ± 0.16 ^b^	1.07 ± 0.02 ^a^
	sesquiterpene hydrocarbon	24.75	1625	*α*-cubenene	0.09 ± 0.01 ^a^	0.04 ± 0.02 ^a^	0.07 ± 0.06 ^a^
	sesquiterpene hydrocarbon	24.89	1634	*α*-cadinene	0.01 ± 0.01 ^a^	0.01 ± 0.01 ^a^	0.03 ± 0.01 ^a^
	sesquiterpene hydrocarbon	22.29	1463	aromadendrene	0.24 ± 0.05 ^a^	0.2 ± 0.04 ^a^	-
	sesquiterpene hydrocarbon	22.59	1482	alloaromadendrene	-	-	0.48 ± 0.09 ^c^
	sesquiterpene hydrocarbon	23.91	1569	*α*-muurolene	0.84 ± 0.1 ^a^	-	-
	sesquiterpene hydrocarbon	24.02	1576	*γ*-muurolene	0.24 ± 0.02 ^a^	0.47 ± 0.39 ^a^	-
	sesquiterpene hydrocarbon	24.28	1593	zonarene	0.06 ± 0.01 ^a^	-	-
	sesquiterpenic alcohol	18.09	1202	linalool	8.26 ± 1.04 ^ab^	7.67 ± 0.13 ^bc^	6.58 ± 0.23 ^c^
	sesquiterpenic alcohol	20.25	1335	geraniol	0.05 ± 0.01 ^a^	-	0.05 ± 0.02 ^a^
	sesquiterpenic alcohol	26.92	1774	farnesol	1.46 ± 0.27 ^a^	0.63 ± 0.02 ^b^	1.19 ± 0.17 ^a^
	sesquiterpenic alcohol	26.36	1735	*τ*-cadinol	0.85 ± 0.19 ^a^	0.32 ± 0.04 ^b^	0.61 ± 0.06 ^a^
	sesquiterpenic alcohol	26.55	1748	*τ*-muurolol	0.93 ± 0.2 ^a^	0.37 ± 0.02 ^b^	0.63 ± 0.06 ^a^
	sesquiterpenic alcohol	25.69	1689	guaiol	0.22 ± 0.02 ^a^	0.22 ± 0.02 ^a^	0.35 ± 0.03 ^b^
	sesquiterpenic alcohol	26.19	1723	epi-cubenol	-	0.07 ± 0.02 ^b^	0.16 ± 0.01 ^c^
	sesquiterpenic alcohol	25.88	1701	junenol	0.15 ± 0.03 ^a^	-	-
	sesquiterpenic alcohol	25.08	1647	*α*-elemol	0.05 ± 0.01 ^a^	0.05 ± 0.01 ^a^	0.05 ± 0 ^a^
	sesquiterpenic alcohol	25.5	1676	germacrene-D-4-ol	-	-	0.22 ± 0.04 ^c^
	sesquiterpenic alcohol	25.74	1692	caryophyllene alcohol	0.1 ± 0.01 ^a^	-	-
	sesquiterpenic alcohol	25.17	1653	10-epi- γ -eudesmol	0.07 ± 0.03 ^a^	-	-
	sesquiterpenic alcohol	26.71	1759	agarospirol	-	-	0.08 ± 0.02 ^c^
	sesquiterpenic alcohol	26.59	1751	bulnesol	0.08 ± 0.02 ^a^	-	-
	sesquiterpenic alcohol	27.03	1137	β-acorenol	-	0.05 ± 0.01 ^a^	-
	sesquiterpenic alcohol	27.15	1790	cubebol	0.01 ± 0.01 ^a^	-	-
	sesquiterpenic alcohol	20.76	1367	nerol	-	0.02 ± 0.01 ^a^	-
	monoterpene ester	20.14	1329	linalyl acetate	-	-	0.01 ± 0.01 ^c^
	monoterpene ester	21.95	1442	geranyl Acetate	1.02 ± 0.03 ^a^	0.69 ± 0.07 ^b^	1.01 ± 0.05 ^a^
	monoterpene oxide	17.30	1154	eucalyptol	0.04 ± 0.01 ^a^	0.07 ± 0.01 ^a^	0.04 ± 0.02 ^a^
Total aliphatic derivatives					5.5± 0.72 ^a^	6.52± 1.13 ^a^	4.57± 0.61 ^a^
	ester	28.32	1873	(E,E)-farnesyl acetate	0.76 ± 0.2 ^a^	0.35 ± 0.02 ^b^	1.08 ± 0.2 ^a^
	ester	15.04	1015	3-methyl-3-butene-1-ol acetate	1.27 ± 0.14 ^a^	1.22 ± 0.02 ^a^	0.61 ± 0.06 ^b^
	ester	15.51	1044	prenyl acetate	2.57 ± 0.29 ^a^	4.23 ± 0.06 ^b^	2.15 ± 0.11 ^a^
	ester	16.53	1106	cis-3-hexenyl-acetate	0.04 ± 0 ^a^	0.04 ± 0.01 ^a^	0.06 ± 0.03 ^a^
	ester	14.17	960	isobutyl acetate	-	0.01 ± 0.01 ^a^	-
	ester	13.94	945	butyl acetate	0.09 ± 0.03 ^a^	0.07 ± 0.03 ^a^	-
	ester	16.72	1118	hexyl-acetate	0.49 ± 0.1 ^a^	0.49 ± 0.03 ^a^	0.44 ± 0.05 ^a^
	ester	14.77	999	1-butanol, 3-methyl, acetate	-	-	0.11 ± 0.03 ^c^
	alcohol	12.42	837	3-buten-2-ol, 2-methyl	0.09 ± 0.02 ^a^	-	-
	alcohol	29.00	1920	1-hexadecanol	-	-	0.06 ± 0.01 ^c^
	alcohol	13.35	907	prenol	0.03 ± 0.03 ^a^	-	-
	alcohol	19.90	1314	citronellol	-	-	0.03 ± 0.01 ^c^
	aldehyde	14.12	957	2-hexenal, (E)	0.14 ± 0.03 ^a^	0.1 ± 0.03 ^a^	0.03 ± 0.01 ^b^
	aldehyde	12.70	859	pentanal	0.02 ± 0.01 ^ab^	0.01 ± 0.01 ^bc^	-
Total (%)					99.52	99.97	99.96

RI = Retention index relative to C7-C28 n-alkanes on Elite-5MS column, MS = (GC/MS). Values are means ± standard errors of means. Relative percentages were calculated from peak areas (peak area relative to the total peak area, %) and were averages of three extractions per method. Means followed by a different letter within the column are significantly different (*p* ≤ 0.05).

**Table 3:** Total content in oxygenated compounds, terpene hydrocarbons, and aromatics of essential oils obtained from ylang-ylang flowers by HD, SFME, and SD.

Extraction Method	Light Oxygenated Compounds	Heavy Oxygenated Compounds	Terpene Hydrocarbons	Aromatics
HD	69.94 ± 7.53 ^abc^	13.69 ± 2.90 ^ac^	16.1 ± 1.04 ^ab^	47.45 ± 4.7 ^abc^
SFME	81.23 ± 8.49 ^b^	7.75 ± 0.42 ^b^	10.95 ± 2.71 ^b^	53.4 ± 2.90 ^b^
SD	57.98 ± 6.62 ^c^	13.68 ± 1.72 ^c^	27.12 ± 2.05 ^c^	41.66 ± 3.6 ^c^

Values are means ± standard errors of the mean and were averages of three extractions per method. Means followed by a different letter within the lines are significantly different (*p* ≤ 0.05), Tukey’s test.

## Data Availability

The original contributions presented in this study are included in the article. Further inquiries can be directed to the corresponding author.

## Appendix A

**Table A1:** Comparison of EOs from this study with international AFNOR standards [21].

Parameter	AFNOR Extra-Sup (Comoros)	AFNOR Extra (Comoros/Madagascar)	AFNOR First (Comoros/Madagascar)	AFNOR Second (Comoros/Madagascar)	AFNOR Third (Comoros/Madagascar)	HD Extract	SFME Extract	SD Extract
Relative density at 20 °C	0.970–0.990	0.955–0.976/0.950–0.965	0.938–0.960/0.933–0.949	0.925–0.945/0.922–0.942	0.906–0.925/0.906–0.925	0.985	0.988	0.959
**Components**								
prenyl acetate	1.5–3.2	1–2.3/0.6–2.2	0.3–1.8/0.2–1	0.2–0.9/0.1–0.5	0.1–0.2/Traces–0.2	2.57	4.23	2.15
*p*-cresyl methyl ether	7–13	5–13/7–16	3–8.5/5–10	2–5/1–4.6	0.1–1/0.1–1.4	12.62	13.54	11.23
methyl benzoate	4.5–8	4–6.5/4.5–9	1.5–5.5/3–5	1–3.5/1–3	0.1–0.8/0.1–0.9	8.39	9.01	6.23
linalool	8–13	7–12/15.24	3–10/12–19	2–6/4–9.5	0.1–2/0.6–4	8.26	7.67	6.58
benzyl acetate	14–20	11–17.5/5.5–14	6–14/2.8–10	4–8.8/0.5–5	0.5–3/0.1–2.2	20.14	28.94	15.98
geraniol	0.1–0.7	0.1–0.5/1.3–3	0.1–0.3/1.6–2.6	0.1–0.3/0.7–2.4	Traces–0.1/0.2–0.8	0.05	ND	0.05
geranyl acetate	2–6	2.5–6/7.0–14	2–5/8–15	1.7–6/5.6–12	0.4–3/1–6.6	1.02	0.69	1.01
E-cinnamyl acetate	4–6	3–6.5/0.5–3	2.2–5/0.5–2	2–4.8/0.4–2.2	0.5–2.5/0.1–2	7.48	7.84	7.06
β-caryophyllene	2–6	2.5–8/2.5–8.5	4–10/5.5–12	4.8–14/10–17	5–15/12–19	ND	ND	ND
germacrene D	9–15	14–20/5–15	10–24/9.5–18	16–28/13–28	20–35/15–34	5.8	3.55	12.24
(E,E)-α-farnesene	2–6	6.5–15/1–5	7–18/3–8	14–21/5–11.5	12–29/9–25	7.15	5.68	12.61
(E,E)-farnesol	0.8–1.5	0.8–1.6/0.5–3	0.8–2/0.1–2.5	0.8–3/1.2–3.5	0.8–3/1.2–4	1.46	0.63	1.19
benzyl benzoate	3–6	4–6/3.5–8	4.2–9.2/4.5–8	4.5–7.8/6–10	4–8/4.8–8.5	7.43	4.6	7.08
(E,E)-farnesyl acetate	1–3	1–3/0.5–3	1–4/1–2	1–3.5/1.2–3.5	1.5–5/1.7–5	0.76	0.35	1.08
benzyl salicylate	1.5–3.5	2–3.8/1.2–4	2–4/1.6–4	2–4/1.8–4	2.5–4.8/2–5	3.16	1.73	4.6
